# Spontaneous torsion of the right upper lung lobe: a case report

**DOI:** 10.1186/s40792-017-0313-3

**Published:** 2017-02-22

**Authors:** Yusuke Kita, Tetsuhiko Go, Kazuhito Nii, Natsumi Matsuura, Hiroyasu Yokomise

**Affiliations:** Department of General Thoracic, Breast and Endocrinological Surgery, Kagawa University Hospital, 1750-1 Ikenobe, Miki-cho, Kida-gun, Kagawa 761-0793 Japan

**Keywords:** Spontaneous torsion, Lung cancer, Pleural effusion, Emergency lobectomy

## Abstract

**Background:**

Pulmonary torsion is usually caused by thoracic surgery or trauma. Spontaneous pulmonary torsion caused by tumor and pleural effusion is very rare.

**Case presentation:**

A 76-year-old Asian male with a chronic cough and suspected lung or pleural tumor presented with sudden dyspnea. Computed tomography showed that the right upper lung lobe contained a large tumor in the region of S1-3; the tumor had shifted to the posterior thoracic space and rotated 90° counterclockwise, potentially impeding blood flow. The patient underwent emergency right upper lobectomy for torsion of the right upper lung lobe. He recovered uneventfully and was discharged without complications.

**Conclusions:**

We experienced a rare case of spontaneous torsion of the right upper lung lobe caused by a large tumor and massive pleural effusion.

## Background

Pulmonary torsion is usually caused by thoracic surgery or trauma. Spontaneous pulmonary torsion caused by tumor and pleural effusion is very rare [1], and is defined as parenchymal rotation on the bronchovascular pedicle [2]. This rotation of the bronchovascular pedicle means that life-saving emergency surgery is required.

## Case presentation

**Fig. 1:**
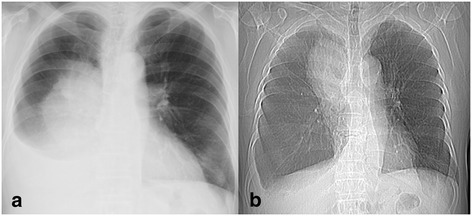
**a** Chest radiograph showing a large tumor shadow in the middle lung field and pleural effusion in the right cavity. **b** Scout image showing a large tumor shadow in the upper lung field. Note the change in position of the tumor

**Fig. 2:**
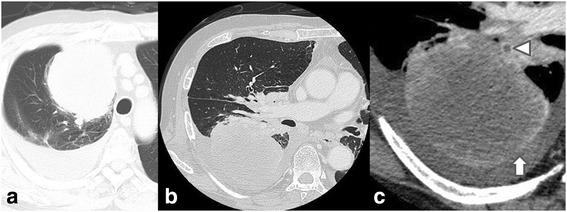
**a** Plain CT at first visit showing a large tumor mainly located at right S1-3, widely in contact with the precordial mediastinum and pleura. Massive pleural effusion in the right thoracic cavity is also present. Contrast-enhanced CT image at the onset of torsion, **b** lung window, **c** mediastinal window (enlarged image). The tumor in the right upper lung lobe has moved to the posterior thoracic space. There is a thrombus in the upper pulmonary vein (*triangle*) and non-contrast regions in the peripheral tumor (*upward arrow*), leading to suspicion of blood flow impediment

A 76-year-old Asian male had a history of chronic coughing. A chest radiograph taken with the patient in standing position showed pleural effusion and tumor shadows in the right middle lung field (Fig. 1a). Scout imaging revealed that the tumor was located in the upper lung field, with a possibility that it had moved (Fig. 1b). Computed tomography (CT) showed a 13-cm tumor in his right upper lung lobe mainly located at S1-3 and massive pleural effusion in the right thoracic cavity (Fig. 2a). The patient was scheduled for investigation of suspected lung cancer or pleural tumor.

**Fig. 3:**
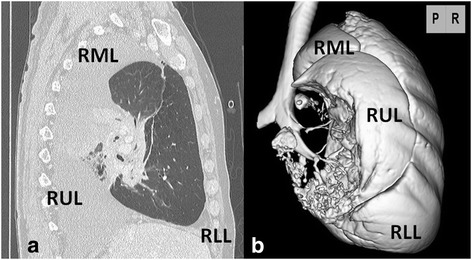
**a** Sagittal CT at the onset of torsion showed that the right upper lung lobe with the large tumor had rotated 90° counterclockwise toward the hilum. **b** Three-dimensional CT by the view of posterior showed that the middle and lower lung lobes had been pushed upward and forward respectively. The *blank space* showed a tumor. *RUL* right upper lobe, *RML* right middle lobe, *RLL* right lower lobe

Twelve hours later, he came to ER with sudden dyspnea and fever. Vital signs were blood pressure 156/87 mmHg, heart rate 117 bpm, temperature 39.0 °C, respiratory rate 20 breaths/min, and percutaneous oxygen saturation of 96% on 5 L oxygen flow. CT showed that the right upper lung lobe had shifted to the posterior thoracic space (Fig. 2b, c), causing the right middle and lower lung lobes to be pushed up toward to the apex; the right upper lung lobe had also rotated 90° counterclockwise toward the hilum (Fig. 3a, b). The pulmonary artery was not disrupted but was stretched in an arc. There was a thrombus in the upper pulmonary vein and non-contrast regions in the peripheral tumor; impediment to blood flow was suspected. Torsion of the right upper lung lobe was diagnosed, and emergency right upper lobectomy was performed through a right anteroaxillary thoracotomy.

Intraoperatively, the right upper lung lobe had shifted to the posterior thoracic space; the middle lobe was compressed to the apex; and the lower lobe was distended in the hilar front. The right lung had rotated 90° counterclockwise toward the hilum. There was no color change of the pleura due to ischemia and congestion from the lung surface. There was about 1300 ml of serous pleural effusion, which was no malignant. The pulmonary vessels had been stretched but were not completely obstructed. There were no adhesions in thoracic space, but the oblique and horizontal fissures of the lung were incomplete by thickening of pleura due to inflammation. As concerned about thrombus and necrotic material in the right upper lung lobe, we first dissected the pulmonary vein, then the superior trunk of the pulmonary artery, before resolving the torsion and performing the lobectomy.

The patient recovered uneventfully and was discharged. Pathological examination of the resected lung revealed a lung adenocarcinoma, pT3N2M0 stage IIIA. The patient is undergoing postoperative chemotherapy.

## Discussion

Pulmonary torsion is rare and is usually caused by thoracic surgery or trauma. Ohde and colleagues wrote that approximately 10 cases of spontaneous pulmonary torsion have been reported in English literature to date [1]. But the frequency with which spontaneous torsion of an entire lung occurs is somewhat difficult to assess for did not state the clinical setting of each case. Irie and colleagues wrote only 3 cases of entire lung torsions that spontaneously occurred have been reported in English literature since 1988 [2]. Since then, to our knowledge, only 5 cases of entire lung lobe torsion in English [1–5] reported causes of spontaneous pulmonary torsion include pulmonary tumor, atelectasis, pneumothorax, infection, and pleural effusion. The lung torsion in our case was caused by a large tumor and massive pleural effusion, which is very rare.

CT is the best single diagnostic test for lung lobe torsion [6]. CT enabled prompt diagnosis of lung torsion in our case. Radiographic signs of pulmonary torsion include change in position of an opacified lobe and hilar displacement [7]. In our case, comparison of the Scout image and chest radiograph would have showed that the location of the tumor had changed. Although it is not conclusive for shooting condition differ between standing position and supine position, because the tumor position of the image was moved, the possibility of torsion should be considered. We could potentially have prevented torsion by immediate drainage or paracentesis of the pleural effusion, since the large tumor and pleural effusion are speculated to be a cause of spontaneous lung torsion and change in position of the lobe were detected [4, 7].

Urgent intervention is required for this disease because of the high risk of mortality related to thrombus, necrotic material, and infection of the lobe if treatment is delayed [1, 8]. In our case, CT showed a thrombus in the upper pulmonary vein; however, the patient did not develop thrombosis because we resected the pulmonary vessel before conducting the lobectomy.

## Conclusions

In conclusion, spontaneous lung lobe torsion needs to be diagnosed and managed promptly. Emergent surgery may be mandatory to save the patient. Early phase drainage may prevent spontaneous pulmonary torsion in case of large tumor with massive pleural effusion.

## Abbreviations

CT: Computed tomography
RLL: Right lower lobe
RML: Right middle lobe
RUL: Right upper lobe
